# Monitoring and Control of the Direct Energy Deposition (DED) Additive Manufacturing Process Using Deep Learning Techniques: A Review

**DOI:** 10.3390/ma19010089

**Published:** 2025-12-25

**Authors:** Yonghui Liu, Haonan Ren, Qi Zhang, Peng Yuan, Hui Ma, Yanfeng Li, Yin Zhang, Jiawei Ning

**Affiliations:** 1 College of Engineering, Ocean University of China, Qingdao 266400, China; liuyonghui@ouc.edu.cn (Y.L.); rhn0302@outlook.com (H.R.);; 2 Shandong Key Laboratory of Additive Manufacturing Technology & Equipment, Ocean University of China, Qingdao 266400, China; 3 Shandong Liwei Laser Technology Co., Ltd., Taian 271208, China; 4 College of Mechanical and Electronic Engineering, China University of Petroleum, Qingdao 266580, China; 5 Shandong Huayun 3D Technology Co., Ltd., Jinan 250098, China

**Keywords:** additive manufacturing, directed energy deposition, deep learning, in situ monitoring, process control

## Abstract

Directed Energy Deposition (DED), as a core branch of additive manufacturing, encompasses two typical processes: laser directed energy deposition (LDED) and wire and arc additive manufacturing (WAAM), which are widely used in manufacturing aerospace engine blades and core components of high-end equipment. In recent years, with the increasing adoption of deep learning (DL) technologies, the research focus in DED has gradually shifted from traditional “process parameter optimization” to “AI-driven process optimization” and “online real-time monitoring”. Given the complex and distinct influence mechanisms of key parameters (such as laser power/arc current, scanning/travel speed) on melt pool behavior and forming quality in the two processes, the introduction of artificial intelligence to address both common and specific issues has become particularly necessary. This review systematically summarizes the application of DL techniques in both types of DED processes. It begins by outlining DL frameworks, such as artificial neural networks (ANNs), recurrent neural networks (RNNs), convolutional neural networks (CNNs), and reinforcement learning (RL), and their compatibility with DED data. Subsequently, it compares the application scenarios, monitoring accuracy, and applicability of AI in DED process monitoring across multiple dimensions, including process parameters, optical, thermal fields, acoustic signals, and multi-sensor fusion. The review further explores the potential and value of DL in closed-loop parameter adjustment and reinforcement learning control. Finally, it addresses current bottlenecks such as data quality and model interpretability, and outlines future research directions, aiming to provide theoretical and engineering references for the intelligent upgrade and quality improvement of both DED processes.

## 1. Introduction

Additive manufacturing (AM) is considered one of the most disruptive technologies due to its unique manufacturing capabilities, such as high utilization rate of material, design freedom, ability to manufacture complex components, etc. [1].

Over the past several decades, with the continuous advancement of technology, especially the continuous maturity of metal AM technology, AM technology has gradually expanded from the initial rapid prototyping tools to terminal manufacturing technology with broad application prospects in fields such as aerospace, biomedicine, and personalized manufacturing [2,3,4,5,6]. According to the Wohlers 2025 Report on AM, in 2024, the AM industry grew 9.1%, or nearly $21.9 billion, and the total AM market is expected to reach $115 billion by 2034 [7]. Directed energy deposition (DED), as an important branch of metal AM, fuses metal powder or wire by focused thermal energy (such as laser, electron beam or plasma arc), deposits the melted material along a predetermined path, and constructs parts layer by layer [8]. The importance of DED to industry in its current state is highlighted by three-fold application of cladding, repair, and construction, although it does have some drawbacks, such as poor dimensional tolerances, poor surface quality, and limited ability to produce complex shapes. DED encompasses two typical processes: laser directed energy deposition (LDED) and wire and arc additive manufacturing (WAAM), which are widely used in manufacturing aerospace engine blades and core components of high-end equipment [9,10,11].

**Figure 1:**
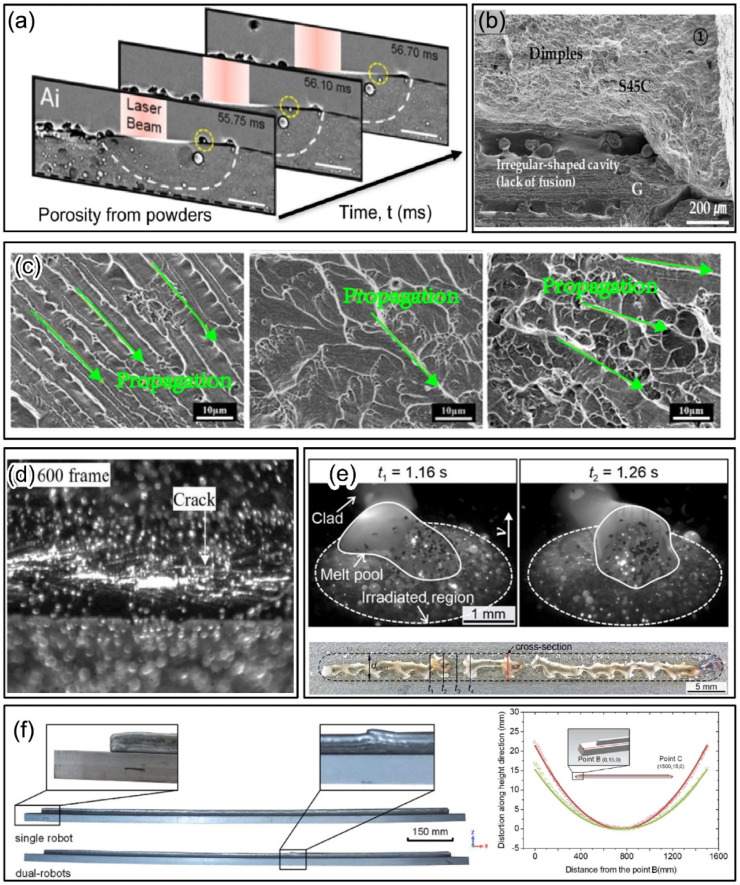
Summary of typical process-induced defects in DED (**a**) Porosity in LDED [14]. (**b**) Lack of fusion in DED [17]. (**c**) Microstructural heterogeneities in WAAM [20]. (**d**) Cracking in LDED [21]. (**e**) Balling in LDED [22]. (**f**) Distortion in WAAM [23].

However, metal AM is a technology that involves complex interactions between the heat source, materials, substrate, and environment, with these factors being highly dynamic and interdependent [12]. As a result, various defects are prone to occur during the AM process, which may cause the strength, toughness, damage tolerance, and fatigue performance of the components fabricated by metal AM be lower compared with traditional technologies such as forging [13]. This presents a challenge to the widespread application of AM, especially for a large structural part in high-end industrial fields such as aerospace. Thus, measures must be taken to minimize the occurrence of defects in practical applications. Some of the typical process-induced defects in DED are summarised in Figure 1.

Porosity is one of the most common and significant defects in AM, with a notable detrimental impact on the mechanical properties of the parts, especially fatigue performance [14,15]. The formation of porosity can result from various causes, including the evaporation of low-melting-point elements in alloys, keyhole defects caused by excessive energy density, and gas inclusions in the raw materials. Lack of fusion defects are generally caused by insufficient heat input. When the heat source power is set incorrectly, the scanning speed is too fast, or the layer height is inappropriate, the heat input per unit of time is insufficient, preventing the metal from fully melting, thus leading to lack of fusion defects [16,17]. During subsequent deposition, these unmerged areas may cause irregular porosity formation, even affecting the deposition quality of the next layer. In AM, even minor process changes can lead to uneven distribution of the material’s microstructure, manifested as inconsistent shapes, sizes, orientations, and elemental distribution of the grains. Many studies have explored the relationship between this microstructural inhomogeneity and the mechanical properties of the material, such as tensile properties, hardness, and fracture toughness [18,19,20]. Due to stress concentration and the presence of a liquid film during solidification, cracks may form at high-angle grain boundaries [21]. Furthermore, during the heating process of metal powders, if they do not transition smoothly or ideally into spherical structures, they often form irregular or incomplete spherical particles, a condition known as balling defects [22]. Spheroidization defects are typically influenced by multiple factors, including insufficient energy input, inappropriate scanning strategies, and the characteristics of the powder itself. These defects can result in reduced powder flowability, increased surface roughness, and reduced production efficiency. In the AM process, due to the rapid cooling and heating effects of the heat source, significant temperature gradients often develop within the part. The uneven shrinkage caused by these temperature differences can accumulate residual stresses in the part. Residual stresses can lead to deformation, delamination, and even cracking of the part, and may also prevent the final product from meeting design requirements, leading to part failure [23,24,25].

How to find these defects in advance and ensure part quality and repeatability of manufacturing has become one of the biggest problems faced by AM [26,27,28]. There are usually two approaches for the detection of process-induced defects. One is known as ex situ or post-mortem approach which relies on post-production inspection and can be carried out by destructive and non-destructive testing methods [29], such as X-ray Computed Tomography (XCT). The other is called in situ or process-level monitoring approach. Compared with traditional ex situ quality assurance approach based on final state detection, in situ monitoring can achieve early identification and warning of defects, provide feedback for subsequent process parameter regulation and closed-loop control, and thus is the core of building an intelligent AM system [30].

In order to effectively monitor the AM process and control the quality of the part being manufactured, in situ monitoring technology includes three procedures as follows. Firstly, appropriate sensors are utilized to collect AM process signals (such as thermal signal, acoustic signal and optical signal), and obtain real-time status data of the manufacturing process. Secondly, appropriate techniques are utilized to analyse the large amounts of data generated by in situ monitoring of the metal AM process, identify defects and predict quality, and establish the relationship between process parameters and part quality. Finally, process parameters are optimized based on the model prediction results in order to realize the closed-loop process control and the improvement of part quality. Among them, the massive data generated during the monitoring process and the complex relationship between process, signal, and part quality have posed significant challenges to traditional statistic-based signal processing methods.

In order to tackle these difficulties, intelligent monitoring and control of metal AM process using deep learning (DL) techniques have become a hotspot and frontier of research in recent years [9]. With the rapid development of artificial intelligence (AI) technology, machine learning (ML), especially DL algorithms [17,31] are beginning to be utilized for defect detection and quality prediction in metal AM. These algorithms can effectively process the large amounts of data obtained by in situ monitoring of the AM process and help to disclose the relationships between process parameters and part quality.

In recent years, some scholars have published multiple review papers related to AI-based monitoring and control in metal AM. Herzog et al. [9] comprehensively reviewed the process monitoring technologies for laser-based metal AM, the application of ML in defect detection, and the comparison of sensing technologies, with a focus on discussing the future directions and research recommendations for closed-loop process control. Zhang et al. [32] focused on the application of ML in the powder bed fusion (PBF) process, classified sensing methods by spatial scale, systematically summarized defect types, dataset preparation, ML modeling, and feedback control strategies, and analyzed the key challenges in data processing and algorithm optimization. Hamrani et al. [33] conducted a systematic review of the intelligent applications of WAAM through data-driven clustering analysis, covering four major research areas: design optimization, material analysis, defect detection and monitoring, and process modeling and optimization, and clarified the existing problems and opportunities in data, models, and sensing systems. Mattera et al. [34] centered on the intelligent monitoring and control of the WAAM process, detailed the application of DL technologies in defect identification and process parameter monitoring, and emphasized the analysis of multi-source sensing data processing (including arc, acoustic, optical, and thermal signals) and feedback control strategies. The aforementioned literature has reviewed the intelligent monitoring and control of DED from different perspectives such as various AM technologies, sensor application technologies, data processing, and closed-loop process control.

However, there is a lack of s a holistic review on monitoring and control of the DED AM process using DL techniques, such as defect types that can be detected, DL-based sensing signal classification, and the application of DL approaches. To address this, this study conducts an investigation of 270 papers published in the field of AI-based monitoring and control of DED over the past five years, providing a detailed review of the key technologies and research status in the aforementioned aspects. The aim is to systematically review the existing research status of DL applications in the DED field, elucidate the research hotspots in this area, and provide references for scholars in related fields.

During the screening of relevant studies, the systematic review workflow proposed by Xiao et al. [35] was adopted, and a four-step screening protocol was implemented.

(1) Definition of the research question

Based on the current research status of the field and the objectives of this review, three principal research questions (RQs) are specified to guide the full review workflow as follows: Which types of DED problems are currently being addressed by DL? What are types of DL models are employed to tackle DED problems? What are the pros and cons of using DL models to solve DED problems?

(2) Strategy of literature searching

The databases used for literature retrieval include Web of Science, Elsevier, IEEE Xplore, and ScienceDirect. These databases comprehensively cover high-quality scholarly literature relevant to the topic of this review, encompassing journal articles, books, and conference proceedings, thereby mitigating the risk of literature omission from reliance on a single database. Following the decomposition of research questions into core conceptual domains, keywords were combined using Boolean operators: (“directed energy deposition” OR “laser directed energy deposition “OR “wire and arc additive manufacturing “OR “DED” OR “WAAM”) AND (“artificial intelligence” OR “deep learning” OR “neural network”) AND (“defect detection” OR “online monitoring” OR “real-time monitoring”) AND (“closed-loop control” OR “process optimization”).

(3) Criteria for inclusion and exclusion

Inclusion criteria included strong relevance to the study topic and RQs, publication within the past five years, study type being original research articles or review papers, and complete, traceable data; exclusion criteria included conference abstracts or technical reports, the literature unrelated to the topic, and studies with incomplete data.

(4) Quality assessment of the included literature

During the literature screening process, studies were first retrieved via keyword searches. Then the selected studies were filtered according to the aforementioned inclusion and exclusion criteria. Finally, in the quality assessment stage, a scientifically rigorous evaluation was conducted according to the following aspects, i.e., soundness of research design, standardization of data collection, completeness of model validation, and logical coherence of conclusions. After screening, a total of 270 eligible publications were selected as the analysis dataset.

**Figure 2:**
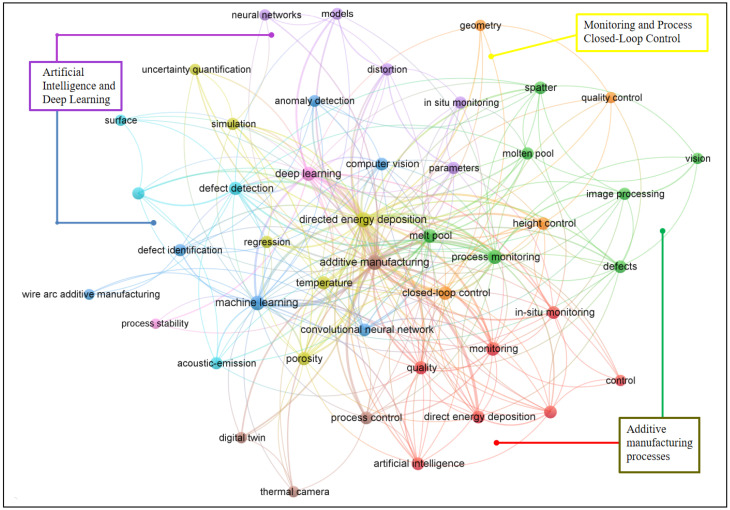
The clustering analysis of the proposed article selection.

The clustering technique, which is based on the co-occurrence matrix [36], was employed in this study to provide insights into emerging research hotspots by analysing keyword distributions in the literature. The clustering technique enables the calculation of the association strength between keywords—where a higher association strength between two keywords indicates a tighter conceptual linkage. Using the visualization results provided by the software of VOSviewer 1.6.20, keyword-level clustering and hotspot identification were achieved. As illustrated in Figure 2, the included studies form five primary clusters, each represented by a distinct color (red, blue, yellow, green, and purple). Under this clustering framework, the spatial proximity between keywords indicates the degree of interrelatedness.

In the yellow cluster, terms such as closed-loop control, geometry, height control, quality control, regression, and simulation are associated with monitoring and closed-loop control techniques in AM processes. DL techniques are primarily represented by the purple cluster, which includes keywords such as neural networks and DL. The purple cluster is closely connected with the blue cluster, which consists of keywords such as machine learning, convolutional neural network, and computer vision. Therefore, these two clusters (blue and purple), which reflect the same research domain but emphasize different aspects, can be merged into a single cluster representing AI technologies in AM. Similarly, the red cluster represented by keywords such as additive manufacturing and directed energy deposition can be merged with the green cluster, which contains terms such as defects, spatter, and melt pool, to collectively represent AM.

Therefore, the clustered keywords were regrouped based on domain understanding, i.e., process, algorithms, and control, so as to represent them more accurately. Three appropriate research domains (RDs) were proposed including additive manufacturing processes (RD1), artificial intelligence and deep learning (RD2), and monitoring and process closed-loop control (RD3), as shown in Table 1.

Using the three research domains derived from keyword clustering, we systematically classified and thoroughly analyzed the 270 key publications. The additive manufacturing process domain (RD1) focuses on the intrinsic characteristics of DED processes, with studies spanning melt-pool dynamics and defect-generation mechanisms. Artificial intelligence and deep learning (RD2), functioning as the algorithmic nucleus of the field, emphasizes the adaptation and optimization of DL models for DED-specific applications. These models not only enable efficient interpretation of RD1 monitoring data but also provide essential algorithmic foundations for RD3 control methodologies. The monitoring and process closed-loop control domain (RD3) is dedicated to developing DL-enabled DED closed-loop control architectures and parameter-optimization strategies, driving DL from theoretical exploration toward engineering deployment and supporting scalable, high-quality DED manufacturing. The literature distribution and content interconnections among the three RDs illustrate the systematic nature of DL applications in DED. RD1 establishes the problem context and data groundwork, RD2 delivers the core algorithmic capabilities, and RD3 realizes the engineering deployment. Together, they make interdependent and stepwise progress, forming the central framework underpinning contemporary research on DL in DED.

According to the above discussion, this work will be organised as follows. “Deep learning” section of this work will present an overview of the most common DL techniques which include discriminative deep learning, generative deep learning and reinforcement learning. “Application of DL technology in DED process monitoring” section will review the technologies currently applied to in situ monitoring of AM processes, grouped by the type of signal to be monitored. This will provide a discussion of sensor implementation, signal resolution and the generated data structures. “DL-based Control of DED Additive Manufacturing” section will present the DL techniques available for applications in process parameter control. “Limitations, Challenges and Future Directions” section will present the limitations and challenges of DL in the application of Directed DED processes, as well as the potential future directions of this technology. Finally, the conclusion is given in “Conclusions” section.

## 2. Deep Learning

**Figure 3:**
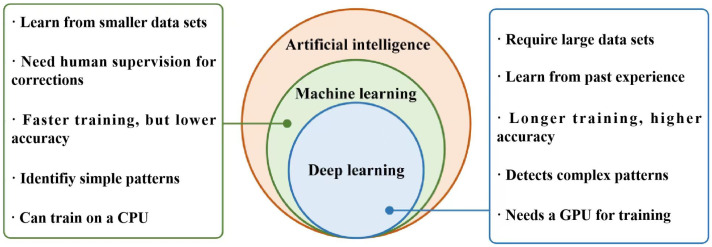
DL and its relationship with ML.

In computer science, AI seeks to create agents capable of perceiving their environment and achieving objectives, with ML as a key subset that traditionally relies on manual feature engineering and data labeling, limiting its ability to capture inherent data features [37]. The transition from ML to DL has been driven by advances in computational power, data scale, and algorithmic innovation, as traditional ML methods with 1-2 hidden layers depend heavily on expert knowledge and struggle with high-dimensional, unstructured data [38]. With powerful hardware—especially GPUs—biologically inspired artificial neural networks were rediscovered and optimized [37,39]. DL uses deep neural networks (≥3 layers) to automatically learn hierarchical feature representations from raw data in an end-to-end manner, reducing reliance on manual feature engineering and emphasizing data quality and hyperparameter tuning [40], as shown in Figure 3. Compared with traditional ML, which mainly captures shallow linear relationships, DL extracts complex nonlinear patterns and intrinsic structures, offering clear advantages for demanding industrial applications such as AM.

**Figure 4:**
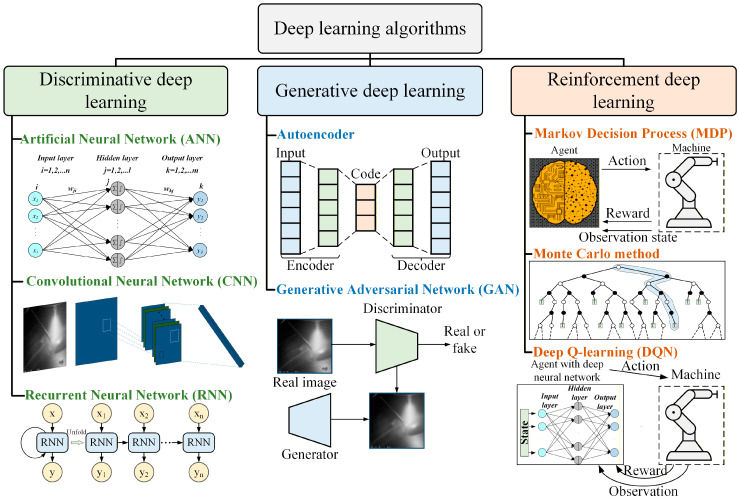
Three types of DL algorithms.

DL efficiently handles large-scale, multimodal sensor data generated in AM processes and achieves high accuracy in microstructure prediction, process control, and defect detection [41,42,43]. Depending on the task, DL algorithms in AM can be categorized into three types, as illustrated in Figure 4.

Discriminative models are used for quality classification, anomaly detection, and process parameter regression [44,45]. Generative models support data augmentation, feature dimensionality reduction, and defect sample generation [30,46]. Reinforcement learning models, employing methods like Markov decision processes and deep Q-learning, enable autonomous real-time control of AM process parameters [47,48].

### 2.1. Discriminative Deep Learning

Discriminative deep learning (DDL) [49] focuses on distinguishing between categories. It processes input data through multiple layers of interconnected neural nodes, learning key features and patterns for classification. As data volume increases, classification accuracy improves. The term “deep” indicates multiple layers, enabling the capture of complex relationships in the data.

#### 2.1.1. Artificial Neural Networks

Artificial neural networks (ANNs), inspired by biological neurons, learn a mapping from input *x* to output *y* by adjusting parameters *θ* to minimize a loss function [50].

(1) $$y=f_{\theta }(x)$$

The universal approximation theorem [51,52] ensures that multilayer neural networks can approximate any continuous function, while backpropagation [53] efficiently optimizes parameters via gradient descent. The basic ANN unit, the neuron, computes a weighted sum of inputs followed by a nonlinear activation.

(2) $$y_{j}=f(\sum_{n}\omega_{j,n}x_{n+b_{j}})$$

Here, $\omega_{j,n}$ is the neuron’s weight vector, $b_{j}$ is the bias, and *f* is the activation function. Neurons are stacked into hidden layers to extract progressively higher-level features for prediction or classification. Activation functions (Sigmoid, Tanh, ReLU, and variants [54]) introduce nonlinearity and influence gradient flow and convergence, while weight initialization (random, Xavier, He, LeCun [55]) prevents vanishing or exploding gradients. Modern DL also uses techniques like batch normalization and residual connections to improve training stability and efficiency.

#### 2.1.2. Convolutional Neural Networks

Convolutional neural networks (CNNs) use convolutional filters to extract hierarchical features from spatially structured data, making them highly effective for image recognition and object detection [56]. To overcome the inability of multilayer perceptrons to capture local correlations, LeCun and Bengio [57] introduced CNNs, enabling end-to-end feature learning. A typical CNN comprises convolutional, pooling, and fully connected layers that progressively derive high-level semantic features from low-level inputs [58]. Numerous architectures have further advanced CNN performance: AlexNet [59], ZF-Net [60], VGG-Net [61], GoogLeNet [62], ResNet [63], and DenseNet [64]. Bianco et al. [65] evaluated CNN accuracy and computational complexity on ImageNet-1k. CNNs are widely applied in AM [66], medical imaging [67], remote sensing [68], and industrial defect detection [69].

#### 2.1.3. Recurrent Neural Networks

Recurrent neural networks (RNNs) [70] are tailored for sequential data, using recurrent connections to retain information from previous inputs and incorporate temporal context into current outputs, making them effective for time-series tasks [68,69]. A single RNN neuron computes:

(3) $$y_{j}^{t}=f(\sum_{n}\omega_{j,n}x_{n}^{t}+b_{j}+\sum_{n}\omega_{j,k}^{r}y_{k}^{t-1})$$

where $\omega^{r}$ is the memory weight matrix, $x_{t}$ is the current input, $b_{j}$ is the bias, and *f* is the activation function. This mechanism models complex temporal dependencies.

RNNs are designed for sequential data, using past information to influence future outputs, making them effective for time-series tasks [71,72]. However, standard RNNs suffer from vanishing and exploding gradients in long sequences [73,74]. To address this, long short-term memory (LSTM) networks [74] and gated recurrent units (GRUs) [75] introduce gated mechanisms-LSTM employs input, forget, and output gates to manage long-term dependencies, while GRU offers a simplified structure with fewer parameters and comparable performance. These models are widely used in time-series prediction, natural language processing, fault diagnosis, and AM process monitoring. For spatiotemporal data, convolutional LSTM (ConvLSTM) incorporates convolution into inputs, hidden states, and gates to capture spatial-temporal correlations [76]. ConvLSTM, using convolution and element-wise multiplication, preserves spatial information while modeling temporal dynamics, making it effective for predicting time-varying patterns. In AM, it has been applied to forecast temperature distributions [77], melt pool geometry and defect evolution [78], and can integrate attention mechanisms [79] to improve long-sequence, spatiotemporal predictions.

### 2.2. Generative Deep Learning

In AM process monitoring, limited high-quality data due to costly materials and experiments can cause supervised models to overfit [80]. Generative deep learning (GDL) mitigates this by enabling unsupervised feature learning, data augmentation, and anomaly reconstruction, modeling data distribution $p_{\theta }\left(x\right)\approx p_{data}\left(x\right)$. It generates new samples resembling real data or extracts latent features *z*, applied in synthetic thermal images [81] and optical image reconstruction [82]. Key GDL structures include autoencoders (AE), generative adversarial networks (GANs), and diffusion models (DM).

#### 2.2.1. Autoencoder

Autoencoders [83] are a typical unsupervised learning model that performs feature extraction and dimensionality reduction by minimizing the difference between the input data xxx and its reconstructed data $\hat{x}$:

(4) $$\hat{x}=f_{dec}\left(f_{enc}\left(x\right)\right)$$

In this case, *f*_enc_ denotes the encoder, which maps the input data to the latent space z, and *f*_dec_ refers to the decoder, which reconstructs the input from z. The optimization goal is typically to minimize the reconstruction error:

(5) $$L={\left||x-\hat{x}|\right|}^{2}$$

In AM, AE are frequently employed for process signal denoising, anomaly detection, and process reconstruction [17,30]. Variational autoencoders (VAE) [84] extend this by modeling the latent variables as probability distributions:

(6) $$L=E_{q_{\phi }(z|x)}\left[logp_{\theta }(x|z)\right]-D_{KL}\left(q_{\phi }(z|x)∥p(z)\right)$$

#### 2.2.2. Generative Adversarial Network

Generative adversarial network [85] generates higher fidelity data by employing adversarial training between the generator G and the discriminator D. The objective function is:

(7) $$\underset{G}{min}\underset{D}{max}V\left(D,G\right)=E_{x∼p_{data}\left(x\right)}\left[logD\left(x\right)\right]+E_{z∼p\left(z\right)}\left[log\left(1-D\left(G\left(z\right)\right)\right)\right]$$

In AM process monitoring, GAN are capable of learning complex process mapping relationships, such as integrating physical information for high-precision process reconstruction [86], and can fuse process simulation with experimental data to predict process anomalies [87].

### 2.3. Reinforcement Learning

Reinforcement learning (RL) is a trial-and-error learning framework where an agent interacts with the environment to learn optimal strategies. At each time step *t*, the agent selects an action *a_t_* based on state *s_t_*, receives a new state *s_t_*_+1_ and immediate reward *r_t_*, and aims to maximize the long-term cumulative reward [88].

(8) $$\underset{\pi }{max}E\left[\sum_{t=0}^{T}\gamma^{t}r_{t}\right]$$

In this context, π(a∣s) denotes the policy function and γ the discount factor. In AM, RL enables real-time decision-making and closed-loop control via a “monitoring-decision-control” framework, leveraging reduced-order models and reward function design for data-driven intelligent control [89]. Early RL methods like Q-learning [90] and SARSA [91] were limited to low-dimensional discrete problems. Deep reinforcement learning (DRL) extends RL to high-dimensional and continuous tasks by using neural networks to approximate value and policy functions. Value-based methods include DQN [92] and double DQN [93], while policy-gradient methods include REINFORCE [94], A3C [95], and PPO [96]. DRL algorithms like DDPG [97] and SAC [98] excel in energy control and multi-parameter collaborative optimization.

## 3. Application of DL Technology in DED Process Monitoring

**Figure 5:**
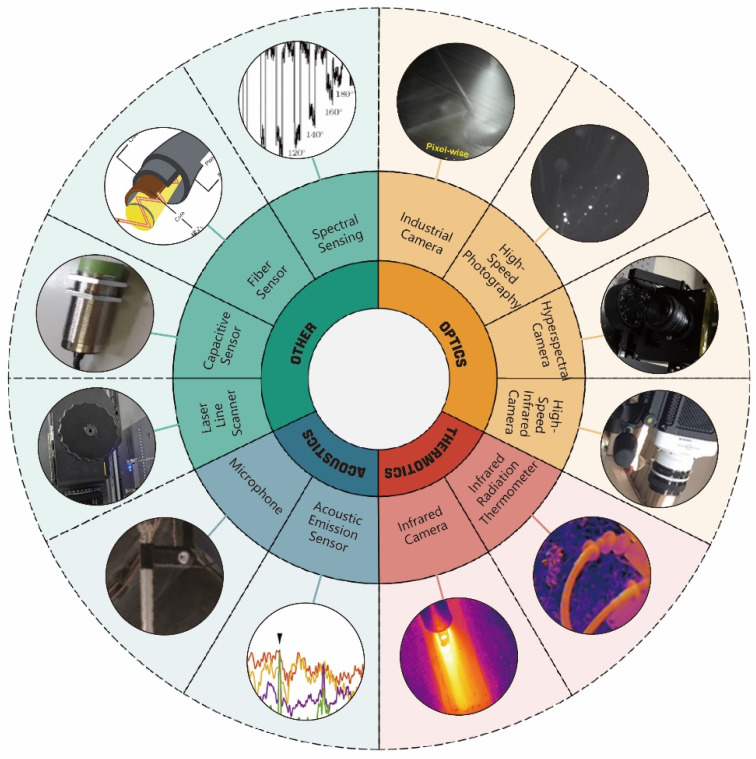
Schematic diagram of common DED process monitoring sensor types and classifications.

In DED metal AM, precise process monitoring serves as the cornerstone for ensuring part quality and enabling intelligent production. This process involves complex multi-field interactions and dynamic changes, where single monitoring dimensions struggle to comprehensively capture molten pool behavior, defect evolution, and quality-related information. Consequently, the industry has developed a multi-dimensional monitoring system integrating optical radiation signals, thermal radiation signals, acoustic signals, and advanced sensing technologies like spectroscopy, fiber optics, and capacitive sensing, as illustrated in Figure 5. DL technology, with its exceptional feature extraction and pattern recognition capabilities, has become a critical tool for analyzing sensor data and uncovering correlations between processes and quality. The following sections will systematically explore the generation mechanisms of different signal types, acquisition technologies, and DL applications, culminating in a multi-sensor fusion strategy that comprehensively demonstrates the technical pathways and core value of DED process monitoring.

### 3.1. DL Applications Based on Optical Radiation Signals

The optical radiation signal serves as a critical physical parameter reflecting both thermal radiation from the molten pool and plasma luminescence behavior during DED processes. In LDED metal AM, optical signal monitoring has become a pivotal technology for process condition monitoring and quality control, thanks to its three core advantages: non-invasiveness, real-time capability, and information richness. This method enables simultaneous capture of molten pool dynamics during part fabrication without contacting high-temperature molten pools or interfering with material deposition processes. It provides multidimensional data including morphology, strength, and spectral characteristics, offering comprehensive support for process condition identification, defect detection, and quality prediction. As such, it forms the core foundation for achieving intelligent DED manufacturing.

#### 3.1.1. Mechanism and Feature Correlation of Optical Signal Generation

The core light signals in metal AM originate from thermal radiation and physicochemical reactions during the interaction between energy sources and materials [99]. In typical processes like laser powder bed fusion (LPBF) and LDED, energy sources such as lasers, electron beams, or plasma arcs irradiate metal powders or wires, rapidly melting them into high-temperature molten pools. The molten pool surface and surrounding areas generate continuous spectral light signals through thermal radiation, with wavelengths spanning from visible light to near-infrared regions [81]. Additionally, characteristic light signals are produced by powder melting splashing, plasma plume formation, and material phase transformations. The intensity, spectral distribution, and dynamic morphology of these signals directly correlate with molten pool temperature, geometric parameters, defects, and microstructural evolution [100].

#### 3.1.2. Optical Signal Acquisition Equipment and Data Characteristics

**Figure 6:**
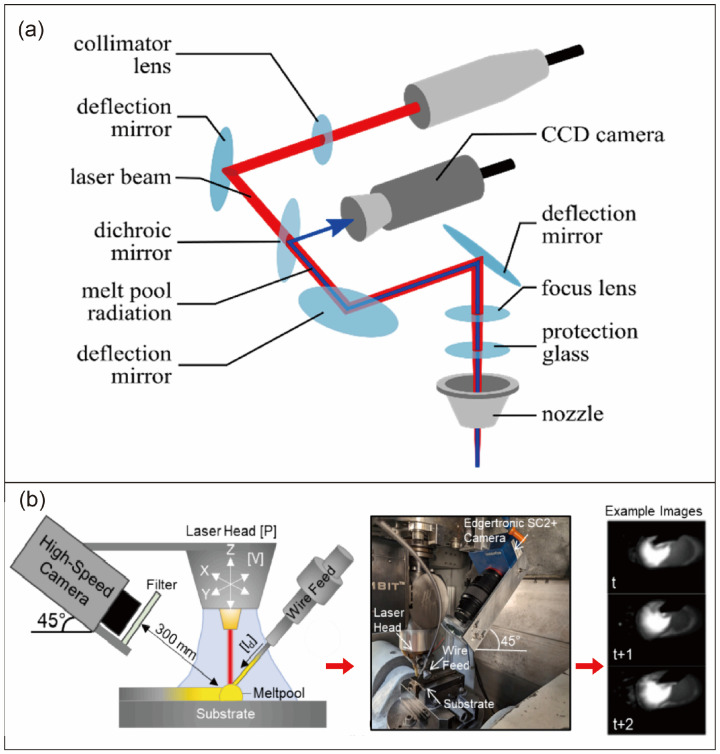
Optical signal monitoring setup (**a**) Optical path of laser beam and melt pool radiation inside the laser head [108]. (**b**) The experimental setup for DED process incorporating off-axial imaging capability and exemplar melt pool data [109].

From a non-invasive perspective, optical signal monitoring utilizes high-spectral cameras and high-speed CCD/CMOS cameras to collect data without contacting the molten pool or altering the processing environment. Optical sensors for light signal acquisition primarily adopt two configurations: coaxial and off-axis setups [101]. The coaxial optical system achieves vertical overhead observation of the molten pool by aligning imaging devices with the laser beam or processing area’s central axis. Snyers et al. [102] employed a near-infrared (NIR) high-spectral camera to coaxially capture 25-band emission signals from the molten pool, forming a high-dimensional data cube to reflect its thermal state. However, coaxial configurations have inherent limitations: difficulty in comprehensively capturing the molten pool’s overall morphology (especially depth information), complex system integration due to strict alignment with the laser axis, and susceptibility to metal spatter and strong reflection interference. In contrast, off-axis optical systems (where imaging devices are positioned at an angle beside the laser beam) offer simpler installation. This approach enables multi-angle data collection of the molten pool and surrounding areas, providing more comprehensive observation with enhanced flexibility and adaptability. Asadi et al. [103] utilized the Cavitar C300 welding camera to record visible/infrared signals from the molten pool at a 45° off-axis angle, achieving a resolution of 1440 × 1080 pixels. However, the oblique viewing angle presents challenges—requiring additional image correction and calibration procedures to accurately restore the actual dimensions of the molten pool, which increases operational complexity and processing time to some extent [104,105,106]. Pandiyan et al. [107] employed a coaxial color CCD camera to capture optical signals in the visible light spectrum in the process zone, effectively avoiding interference from laser-material interactions. This non-invasive approach preserves the molten pool’s native state with precision, providing reliable data for subsequent analysis. It resolves the limitations of contact-based monitoring methods (e.g., thermocouples) that are prone to high-temperature damage and fail to capture dynamic processes, the optical path of the laser beam [108,109] is as shown in Figure 6.

In terms of real-time performance, the high frame rate of optical signal monitoring equipment and the efficiency of data processing models can meet the requirements of closed-loop control in DED processes. For instance, Assad et al. [109] used the Edgertronic SC2 + high-speed camera to capture molten pool optical signals at 2500 fps, enabling the detection of transient anomalies such as droplet ejection and stubbing. Asadi et al. [103] employed the YOLOv8s model, which processes molten pool optical signal images at over 114 fps, delivering real-time segmentation results and geometric parameters. Snyers et al. [102] developed a ResNet-18-based hyperspectral optical signal classification model that completes inference on a single image in just 4.51 ms, significantly faster than the DED molten pool’s dynamic response time constant, thereby supporting real-time anomaly alerts. This real-time capability allows immediate identification of process anomalies, such as molten pool expansion caused by overheating or incomplete fusion due to underheating, creating a critical window for parameter adjustments and preventing defect accumulation.

Information richness constitutes the core competitive advantage of optical signal monitoring. By analyzing optical signals, researchers can obtain multidimensional data on the molten pool’s morphology, intensity, and spectral characteristics. At the morphological level, parameters such as pool area, aspect ratio, and irregularity can be extracted from optical signal images. For instance, stable molten pools exhibit regular elliptical shapes, while the irregularity increases by 36% during stubbing conditions [109]. In terms of intensity, the average optical signal intensity correlates positively with temperature. Under overheating conditions, near-infrared light intensity averages 28% higher than baseline levels, indirectly reflecting energy density variations [101,102]. Spectral level, hyperspectral signals contain 25-band “fingerprint information”, with wavelengths above 820nm contributing over 60% to anomaly classification. Different anomalies, such as overheating or overcooling, correspond to unique spectral signatures [102]. These multidimensional data collectively form a “holistic profile” of process conditions, far surpassing the information dimensions of single-temperature or acoustic signals. Leveraging these advantages, optical signal monitoring plays an irreplaceable role in core DED application scenarios.

#### 3.1.3. Application of DL Technology in Optical Signal Analysis

The core of optical signal processing lies in extracting effective features from raw optical data to achieve quantitative characterization of process status and quality metrics. This field has evolved from traditional image processing to AI-driven approaches. Conventional image processing techniques primarily employ algorithms like filtering, segmentation, and edge extraction for feature extraction, which are suitable for measuring geometric parameters of molten pools in simple scenarios [99]. While these methods demonstrate low computational complexity and fast response times, their performance becomes constrained under complex conditions such as dust obscuration or strong light reflection [110].

With the advancement of AI, DL algorithms have become essential tools in optical signal processing. CNNs and autoencoders have demonstrated remarkable performance in optical signal analysis, particularly excelling in feature extraction, defect classification, and quality prediction under complex conditions [111]. During the LDED process, optical signals are susceptible to interference from dust, ambient light, and equipment noise. Therefore, data augmentation and noise reduction are critical preprocessing steps to enhance signal processing accuracy [112]. In noise reduction, Liu et al. [81] developed an image-enhanced generative adversarial network (IEGAN) based on CNN, which improves image contrast through penalty terms to effectively suppress spatter and background noise. The contrast improvement index (CII) reached 1.1429, surpassing the traditional GAN’s 0.9143. For melt pool segmentation, Mi et al. [113] proposed a deep convolutional neural network that achieves pixel-level semantic segmentation of melt pool and spatter images during LDED, achieving 94.71% accuracy in simultaneous extraction of melt pool morphology and spatter particles. This model maintains high robustness in complex scenarios like strong light interference and powder occlusion, providing a reliable tool for defect identification driven by optical signals. Additionally, Ning et al. [114] combined machine vision with parameter feedback algorithms in thin-walled part LDED forming. By real-time extraction of melt pool contours and establishing mapping relationships between scanning speed and layer height, they achieved highly consistent adaptive compensation, significantly improving forming flatness. In defect detection and classification, Akbari et al. [111] employed ML algorithms including random forest and gradient boosting, combined with optical signal features (e.g., mean intensity and spectral peaks), to achieve multi-classification of molten pool defects with over 8128% accuracy. The algorithm combines a physics model-driven approach for macroscopic contour height instability monitoring and an image data-driven CNN for mesoscopic porosity prediction. Validated on LDED ceramic thin-walled parts, this method achieves 100% recognition of deposition contours and molten pools, effectively capturing micro-scale porosity defects and macro-scale geometric instability—key factors influencing part integrity [115]. This advancement is particularly critical for aerospace components (e.g., engine blades) where micro-defects significantly affect fatigue life, providing a more precise technical means for optical signal-based quality control in high-precision DED scenarios.

### 3.2. Application of DL Technology Based on Thermal Radiation Signal

In LDED or WAAM, thermal signal monitoring is critical for ensuring forming quality. This process fundamentally involves repeated “heating-melting-solidification” cycles, where the uniformity of thermal input directly affects the stability of the molten pool and subsequent solidification behavior. Excessive local thermal input can lead to oversized molten pools and reduced cooling rates, resulting in grain coarsening and element segregation. Conversely, uneven thermal input distribution accumulates significant thermal stress within components, potentially causing defects like warping and hot cracks that severely compromise dimensional accuracy and mechanical properties. Real-time thermal radiation signal acquisition enables effective analysis of temperature field evolution in space and time, visually revealing fluctuations in thermal input uniformity. This provides critical data for evaluating molten pool stability and predicting defect risks [116]. Furthermore, a process condition identification mechanism based on thermal signal feedback drives real-time optimization of key parameters such as laser power and scanning speed [117]. This achieves precise control over thermal input processes, effectively suppressing thermally induced defects at their source, thereby ensuring consistent density and mechanical properties in formed components.

#### 3.2.1. Generation of Thermal Signals

The core thermal signals in metal AM originate from thermal radiation and conduction effects during the interaction between energy sources and materials [118]. In typical processes like LDED, energy sources such as lasers, electron beams, or plasma arcs rapidly melt metal powders or wires, forming high-temperature pools. The molten pool surface generates continuous thermal signals spanning visible to infrared wavelengths through thermal radiation [119]. Additionally, characteristic thermal signals are produced by material melting splashing, plasma formation, and interlayer heat conduction. These signals’ intensity and dynamic variations directly correlate with process parameters, pool conditions, and defects [120].

#### 3.2.2. Thermal Signal Acquisition Technology and Data Characteristics

**Figure 7:**
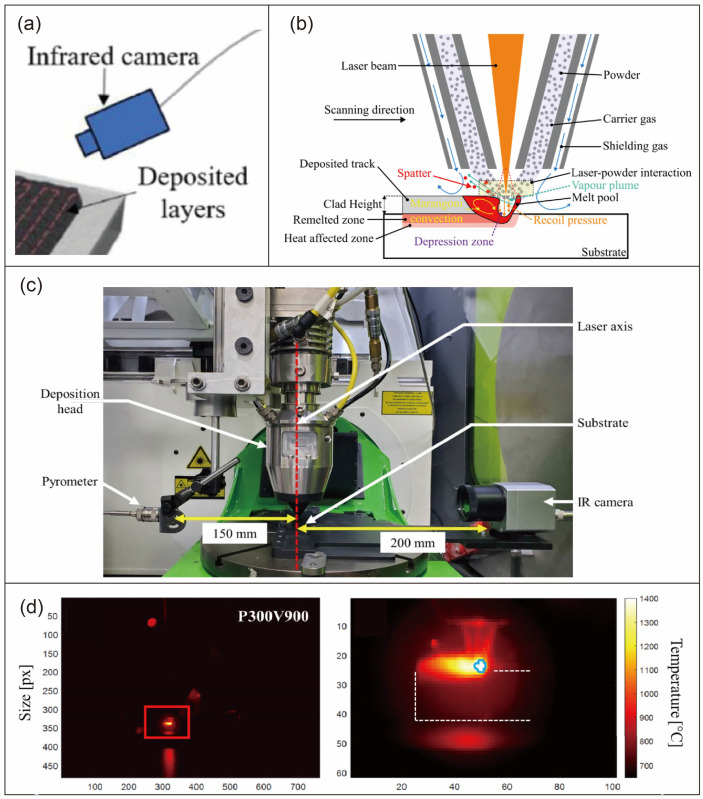
Heat signal monitoring setup (**a**) Infrared camera [121]. (**b**) Schematic of melt pool morphology during DED process and the red dashed line represents the heat distribution of the laser beam in a Gaussian pattern [121]. (**c**) Example of heat signal monitoring setup [122]. (**d**) Images obtained with the IR camera [122].

The core of thermal signal acquisition systems lies in accurately capturing temperature distribution and thermal dynamics, with their performance directly determining the reliability of subsequent analyses. Currently, mainstream acquisition devices and configurations can be categorized into two types: infrared thermal imaging systems and thermal history acquisition systems [121,122], as shown in Figure 7. Infrared thermal imaging systems use infrared cameras as their core components, visualizing temperature fields by capturing thermal radiation signals, making them suitable for real-time monitoring of molten pool morphology and temperature distribution [123]. Thermal history acquisition systems record temporal temperature variations in specific areas using devices like thermocouples and infrared thermometers, ideal for analyzing thermal cycling patterns and cooling rates.

For instance, Jeon I et al. [124] integrated a mid-wave infrared (IR) camera coaxially with the printing laser’s optical path in a DED system. This camera captured melt pool images at 30 Hz to measure melt pool width and length. Features from both devices were input into an artificial neural network (ANN) to estimate melt pool depth online. The total time for measurement and prediction was less than 12 ms, ensuring real-time performance. Kayacan et al. [119] employed an Optris PI 160 infrared camera combined with mathematical modeling to correct measurement errors, enabling precise detection of molten pool temperatures between 1700 and 2800 °C, with 85–98% consistency with finite element analysis results. While these systems offer non-invasive and highly visualized advantages, they are susceptible to environmental radiation and nozzle obstruction interference, requiring specific optical compatibility with the operating environment. Xie et al. [120] utilized a FLIR A655s infrared camera to capture thermal history data of thin-walled components in the DED process at 6–25 fps frame rates, extracting temporal temperature characteristics from 135 regions to provide a foundation for mechanical property prediction. Bertrand et al. [125] coupled a 3D finite element thermal model with the NSGA-II multi-objective genetic algorithm, using experimental single-track deposit geometry and material reference temperatures to identify Goldak heat source parameters, providing reliable data support for LDED process simulation. While this inverse identification method reduces experimental costs and accurately estimates penetration depth, it slightly overestimates HAZ due to coarser meshing in the HAZ region. Additionally, the method relies on post-print geometry observation rather than real-time temperature monitoring, making it unable to capture dynamic thermal field changes during the deposition process, which limits its application in real-time process adjustment.

#### 3.2.3. Application of DL Technology in Thermal Signal Analysis

The core of thermal signal processing lies in extracting effective features from raw temperature data or thermal images to achieve quantitative characterization of process states, evolving from traditional mathematical modeling to AI-driven approaches. Conventional techniques primarily utilize mathematical modeling, noise filtering, and statistical feature extraction to identify critical thermal parameters [126]. Kayacan et al. [119] proposed a temperature calibration model based on weighted averaging, which converts infrared camera measurements into actual pool temperatures. By dividing the area into five temperature sub-regions and accounting for cooling rate effects, the model achieved an error margin of merely 8%.

DL algorithms, with their powerful capabilities in feature extraction and pattern recognition, have become core tools for processing complex thermal signals. Li et al. [127] proposed a CNN-based thermal image classification model that uses LDED process thermal images as input to achieve accurate identification and classification of normal, low-power, and low-speed process states with over 80% accuracy. In melt pool parameter prediction, Ren et al. [128] developed an RNN-DNN hybrid model trained on thermal history datasets generated by finite element simulations, achieving thermal field prediction under arbitrary scanning paths with over 95% consistency with simulation results.

For the prediction of mechanical properties closely related to thermal signals, the microstructure evolution is a key intermediate link. A coupled phase-field and thermal stress dynamics study has shown that in additive manufactured components, precipitate-dislocation interactions are highly sensitive to thermal cycling history, and the heterogeneous distribution of precipitates induced by uneven cooling can lead to a 15–20% difference in local mechanical properties [129]. This physical mechanism can be embedded into physics-informed DL models to improve the interpretability of thermal signal-based mechanical property prediction in DED.

For defect prediction, Xie et al. [120] proposed a wavelet transform-CNN model combining wavelet transform and CNN, converting thermal history signals into time-frequency spectra to predict ultimate tensile strength (UTS) of components with an R^2^ of 0.7, outperforming traditional ML methods. In melt pool segmentation, Liu et al. [81] developed an image enhancement generative adversarial network that improves thermal image contrast through penalty terms, achieving a CII of 1.1429, laying the foundation for melt pool contour extraction. In terms of thermal signal-driven mechanical property prediction, Karthikeyan et al. [130] studied the role of melt pool thermal imaging in DED of 316L stainless steel, which was synchronized with acoustic emission and accelerometer data to capture melt pool temperature variations and spatter events, validating the correlation between high-frequency AE signals and pore formation mechanisms. At the same time, it is clear that the SED of unfused pores, one of the main defect types, is usually larger than 150 μm, and the spatter-induced pore size is related to the powder particle size of 44–106 μm. From the perspective of defect size range, AI can effectively identify pores with SED ≥ 36.3 μm.

### 3.3. AI Monitoring Based on Acoustic Signals

Unlike optical and thermal signals commonly used to monitor external characteristics and changes in the molten pool during the LDED process [131], acoustic detection technologies (including acoustic emission, laser ultrasonics, Rayleigh waves, and guided wave methods) demonstrate unique advantages in identifying internal defects such as cracks, porosity, and incomplete fusion [101]. By capturing broadband transient acoustic signals generated during material deposition, acoustic monitoring provides critical data to understand defect formation mechanisms and assess material integrity.

#### 3.3.1. Sound Signal Generation and Acquisition

The DED process combines WAAM and laser metal deposition (LMD), with acoustic signals exhibiting distinct process-specific characteristics. In WAAM, acoustic emission primarily originates from plasma expansion during arc formation. The volume changes in the ionized zone induce localized pressure fluctuations, generating acoustic signals whose intensity correlates closely with arc dimensions. Conversely, LMD acoustic signals mainly result from laser-powder interaction. When molten powder particles undergo approximately 8.5% volume expansion during melting, subsequent solidification causes contraction. This phase transition disrupts surrounding gases and generates acoustic waves. Additionally, collisions between molten particles during unstable process phases release extra acoustic energy [132].

**Figure 8:**
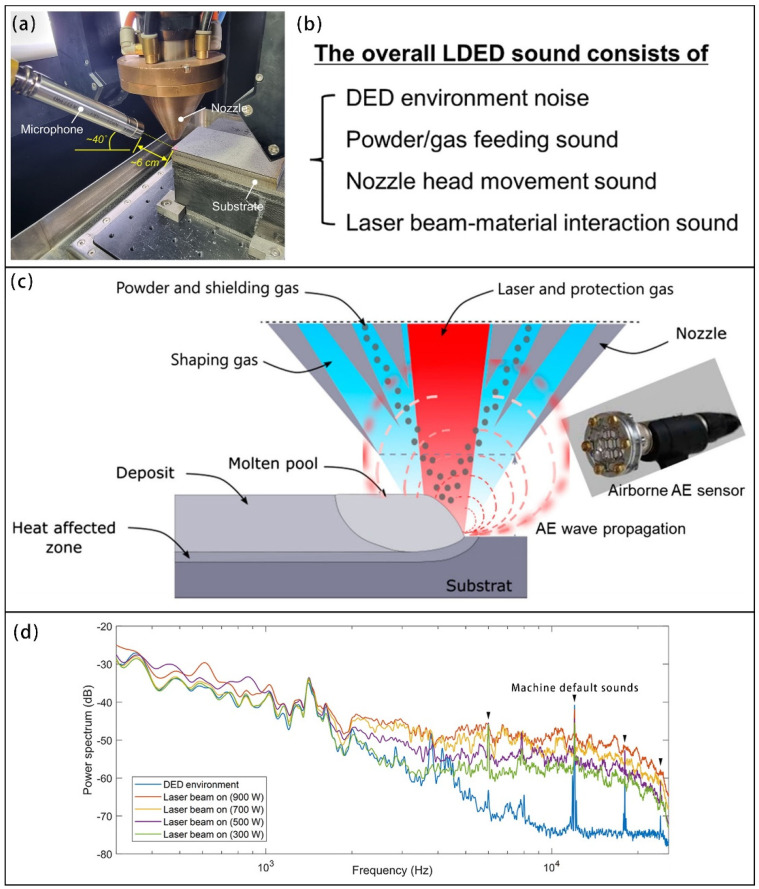
Sound signal acquisition setup (**a**) LDED setup with a microphone installed [133]. (**b**) Identification of sound sources [133]. (**c**) Schematic of the LDED process with an airborne AE sensor and the corresponding AE wave propagation [134]. (**d**) Acoustic signals in the frequency-domain [133].

As shown in Figure 8, the acoustic acquisition system comprises two types of sensors: airborne microphones and contact acoustic emission (AE) sensors [133,134]. Microphones are designed to capture airborne sound signals [135].

#### 3.3.2. Feature Extraction and Data Processing of Sound Signals

The core of acoustic signal processing lies in extracting effective features from raw signals to map them to process states. The acoustic signals from LDED and WAAM processes exhibit non-stationary and multi-component superposition characteristics. Traditional extraction methods primarily rely on filtering, time-frequency analysis, and feature statistics. Filtering effectively suppresses noise, while wavelet transform is widely applied in time-frequency analysis. For instance, Giulio et al. [136] proposed an online monitoring method based on wavelet decomposition and cluster analysis for the working process of the WAAM process. By extracting the time-frequency features of the welding voltage signal and conducting Gaussian mixture model cluster analysis, real-time monitoring of the process status and abnormal early warning were achieved. Kim et al. [133] focused on the crack formation process during LDED, determined the frequency range of crack generation based on the time-domain and frequency-domain signal characteristics of the sound during the forming process, and proposed an effective method for identifying crack generation during the rapid cooling process of LDED.

#### 3.3.3. Application of AI in Sound Signal Defect Detection

Currently, DL algorithms have emerged as a core solution to overcome the limitations of signal analysis, thanks to their powerful data processing capabilities, providing more effective approaches for identifying and processing complex acoustic signals. Hossain et al. [137] proposed an in-situ monitoring method for metal AM processes based on acoustic emission technology, achieving process condition classification through the integration of wavelet transform and CNNs. The method demonstrated 96% classification accuracy on the test set, validating the strong correlation between acoustic emission signals and process conditions.

DL technology demonstrates unique advantages in complex defect identification and noise suppression. Chen et al. [138] collected acoustic data through a robotic LDED system, localized defect areas using optical microscopy, and implemented defect classification by integrating mel-frequency cepstral coefficients (MFCC) features with CNN models, ultimately achieving an overall accuracy of 89% on the denoised dataset. For multi-layer deposition monitoring in WAAM, Rahman et al. [139] discovered through experiments that acoustic emission signals exhibit significant consistency in statistical characteristics during multi-layer deposition processes of different materials. By applying K-means clustering algorithm for unsupervised learning of acoustic signals, they successfully identified five process states including spatter behavior, deposition process, and solidification state, validating the method’s versatility in multi-material and multi-level printing monitoring.

Chen et al. [140] investigated the characteristics of acoustic signals in LPBF processes, employing techniques such as fast fourier transform (FFT) and power spectral density (PSD) analysis to reveal key features—including static pressure fluctuations, standing wave patterns, and high-frequency energy correlated with the amount of melted powder—providing guidance for signal processing in defect-related acoustic monitoring. Wasmer et al. [134] compared seven mainstream AI algorithms and found that optical emission spectroscopy (OES) combined with nonlinear ML models (e.g., multilayer perceptrons and random forests) demonstrated superior performance in functional gradient material (FGM) printing monitoring, achieving classification accuracy exceeding 90%. In contrast, microphone acoustic signals exhibited classification accuracy of only 57.79–78.89% due to high-pressure airflow interference, highlighting the significant advantages of DL over traditional extraction methods. Ansari et al. [141] proposed an AI detection method based on second-order signal derivatives and exponential decay curve fitting. By calculating decay constants, they achieved precise differentiation between cracks and external interference. Furthermore, by correlating laser movement speed with signal peak timing, they successfully localized crack positions.

In AM environments, mechanical noise and gas flow noise can interfere with effective signals, requiring specialized optimization techniques. For the high-noise conditions of DED processes, Chen et al. [142] implemented a three-step method combining audio equalization, bandpass filtering, and harmonic-impulse source separation (HPSS) algorithms to effectively extract the impact-related acoustic components from laser-material interactions. Additionally, an end-to-end framework utilizing fully connected neural networks (FCNNs) and redundant convolutional encoder–decoder (RCED) was developed to minimize environmental noise interference, achieving a root mean square error (RMSE) of 0.81539 on the validation set.

### 3.4. Other

As a core process in metal AM, DED presents significant challenges for monitoring technologies due to its multi-physics field coupling characteristics and dynamic instability. Beyond traditional acoustic, optical, and thermal sensors, emerging technologies like fiber optic sensing, spectral sensing, electrical signal sensing, and vibration sensing have emerged as indispensable solutions. These advanced sensors leverage their unique environmental adaptability and information capture capabilities to enable real-time process monitoring, precise defect identification, and closed-loop quality control.

Spectral sensing technology, serving as a critical tool for monitoring DED processes, provides essential support for process stability evaluation, defect detection, and composition control through its non-contact measurement, rapid response, and elemental analysis capabilities. It primarily comprises two major techniques: laser-induced breakdown spectroscopy (LIBS) and atomic emission spectroscopy (AES). LIBS technology generates plasma through pulsed laser excitation, captures characteristic spectral lines, and combines them with deviation from normal distribution coefficients and standard deviation analysis to achieve rapid quantitative characterization of defects in large-area cladding layers, overcoming the spatial limitations of traditional detection methods. AES directly collects natural emission spectra from the plasma generated during the process of DED, focusing on neutral atomic spectral lines of elements such as Cr, Co, and Mn. Among these, Cr I line appear most frequently and with the highest intensity, while Co I lines and Mn I lines are also detectable. This technology enables real-time correlation between process parameters and process stability, increasing laser power significantly raises the incidence of spectra containing element lines and the intensity of specific Cr I line, while synchronized adjustments of scanning speed and powder feed rate ensure consistent spectral emission characteristics, which reflect process stability [143].

By integrating DL with 3D point cloud data processing, geometric features (e.g., Euclidean distance from the fitted reference plane) enable accurate surface defect detection and localization, achieving an overall recognition accuracy of 91.3% [144]. Although challenges like dust interference and spectral line identification difficulties for low-concentration elements persist, optimized sensor deployment, development of multi-element collaborative algorithms, and multi-modal data fusion will enable spectral sensing technology to achieve seamless integration with DED processing heads, providing more reliable solutions for industrial-grade real-time quality control.

Fiber optic sensors achieve sensing by detecting characteristic changes in light during transmission caused by external physical factors. With their outstanding electromagnetic interference resistance, high-temperature and radiation tolerance, compact size, and support for distributed measurements, they have become the ideal choice for DED processes under extreme conditions. The working principle typically involves embedding optical fibers in structures and combining them with devices like optical time-domain reflectometers (OTDRs) to analyze real-time variations in backscattered light signals. Strain information is obtained through phase shifts caused by axial stretching or compression of the fiber, while temperature is demodulated based on the material’s thermo-optic effect and thermal expansion, enabling simultaneous online monitoring of temperature and strain parameters. These sensors exhibit minimal signal attenuation during long-distance transmission and require no external power supply for the sensing area, making them particularly suitable for DED manufacturing in harsh environments like nuclear reactors. Additionally, distributed fiber optic sensing systems can achieve continuous, high-density data acquisition along the fiber length, with spatial resolution accuracy reaching millimeter-level.

Capacitive sensors (including current and voltage sensors) indirectly monitor arc stability and molten pool conditions during DED processes by tracking welding power supply outputs and circuit signals. The peak and RMS values of current signals reflect arc energy fluctuations, while voltage ripple coefficients correlate with droplet transition patterns. This enables direct correlation between arc energy input/stability and signal fluctuation characteristics. These sensors demonstrate strong compatibility with welding systems, low deployment costs, and signal acquisition without requiring equipment modifications. Furthermore, they can be integrated with power meters and Hall sensors to establish measurement circuits, ensuring both accuracy and synchronization in signal acquisition.

As a core technology in DED process monitoring that combines “active detection” and “dynamic tracking”, laser wire scanning achieves high-precision perception of molten pool morphology, defect distribution, and forming accuracy through controlled laser beam interaction with the processing area. Its key advantage lies in integrating scanning path planning with real-time data feedback, breaking through the spatial limitations of traditional static monitoring to provide comprehensive dynamic evaluation of process conditions. Technically, laser scanning achieves monitoring through two primary methods: First, based on laser triangulation principles, it calculates geometric parameters like molten pool surface height and cladding layer thickness by analyzing angular deviations between emitted beams and reflected light. Second, leveraging laser-induced effects, it modulates laser parameters to induce characteristic responses in the processing area, thereby indirectly reconstructing molten pool temperature and material composition uniformity.

### 3.5. Multi-Sensor Fusion

While single-sensor systems have demonstrated practical value in monitoring DED processes, their inherent sensing principles and measurement characteristics impose significant limitations in comprehensively capturing the complex multi-field coupling dynamics of the process. Single visual sensors can capture geometric information of the molten pool but are prone to interference from splatter and dust obscuration, and fail to directly reflect critical physical parameters such as internal stress and temperature gradients [145]. Acoustic sensors, though capable of detecting vibration and acoustic signals from laser-material interactions, struggle to distinguish defect-induced signals from operational noise, resulting in limited detection of internal defects like micro-pores [146]. Spectral sensors excel at characterizing elemental composition and surface defects, but experience signal instability under extreme temperatures and dusty environments, and lack the capability for simultaneous monitoring of parameters like pool depth and internal stress. Temperature sensors can measure temperature, but contact-type thermocouples may interfere with process operations, while infrared thermal imagers suffer from measurement accuracy limitations due to variations in surface emissivity. Furthermore, research by Chung Baek et al. [147] demonstrates that single-sensor monitoring systems have limited capabilities. These systems cannot effectively address complex state variations caused by multiple interacting factors during DED processes, including process parameter fluctuations, material property changes, and environmental disturbances. For instance, relying solely on electrical signal sensors makes it challenging to establish causal relationships between arc stability and internal defects in the molten pool [148]. Similarly, single geometric monitoring sensors alone cannot comprehensively evaluate the dynamic correlation between melt pool evolution and deposition formation quality in DED processes. These limitations—such as inability to capture real-time thermal-geometry coupling, susceptibility to environmental interference, and failure to distinguish defect causes—highlight the inadequacy of single-sensor signals in fulfilling the requirements for comprehensive DED process monitoring, precise defect diagnosis, and closed-loop quality control. This inadequacy has thereby driven the development of multi-source sensor fusion technologies [149].

#### 3.5.1. Principles and Advantages of Multi-Sensor Fusion

The core of multi-source sensor fusion lies in achieving effective integration and information enhancement of heterogeneous data through rational architectural design and algorithmic processing. Common fusion architectures include data-level, feature-level, and decision-level fusion, with mainstream methods encompassing traditional ML and DL techniques. Data-level fusion refers to directly synchronizing and integrating raw sensor data while preserving complete information, though it requires high data consistency. For example, Kong et al. [149] integrated key multi-modal features into machine learning models for quality prediction. Specifically, the approach extracts frequency-domain energy features from acoustic signals, geometric features of melt pools from visual images through Gaussian filtering and Hough transform, and thermal gradient features from infrared imaging. This represents a typical feature-level fusion strategy, in which discriminative features from each sensing modality are extracted, normalized, and concatenated as inputs to regression models to predict track geometry and defect occurrence. Another feature-level fusion practice combines offline inspection features with online sensor features, employing a locality preserving projection algorithm to reduce dimensionality while maintaining cross-modal correlations.

#### 3.5.2. Application Scenarios of Multi-Sensor Fusion

**Figure 9:**
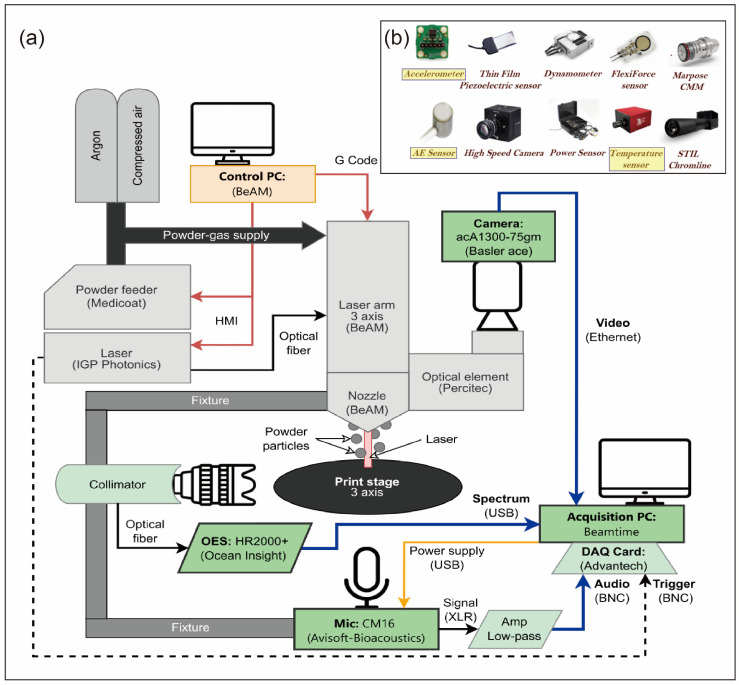
The multi-sensor setup (**a**) The multi-sensor setup of the LDED machine [134]. (**b**) Multimodal sensors array equipped with the DED hybrid machine [152].

DL-powered multimodal sensor fusion has been widely adopted in fields such as AM, autonomous driving, smart home systems, and industrial welding. Its core value lies in addressing complex environmental disturbances and high-precision sensing requirements that single-modal approaches struggle to handle. The DED process involves multi-physics coupling of laser-material interactions, requiring multimodal sensor fusion to simultaneously process data from visual, spectral, acoustic, and electrical signals. As shown in Figure 9, DL technology in this context focuses on two core tasks: defect detection and process parameter optimization.

In defect detection, Guo et al. [150] further introduced a physics-driven deep learning model (PyroNet++) that fuses finite element-simulated molten pool physical characteristics with real-time thermal imaging data. Through collaborative training of CNN and fully connected networks, the MAE for pore size prediction was reduced to 0.0259 mm. For process parameter optimization, Chen et al. [151] designed a Model Predictive Control (MPC) framework based on a time-series dense encoder (TiDE) neural network. The TiDE network simultaneously predicts temporal variations in molten pool temperature and depth over the prediction horizon in a one-shot manner, while integrating multimodal feedback data—including real-time laser power, laser nozzle z-coordinate, and distances from the laser to part boundaries as dynamic covariates—for closed-loop control.

First, the integration of multimodal data enables real-time monitoring of process parameter fluctuations and physical state changes, facilitating stability assessment. In the DED process, Haley et al. [153] combined in situ stereo digital image correlation (DIC) with long-wave infrared (LWIR) thermography to establish a spatial-temporal mapping between melt pool thermal behavior and part deformation. By leveraging natural surface roughness as the DIC pattern, the system avoided process pauses and calibrated infrared temperature readings against thermocouples—obtaining an emissivity of ~0.23–0.25 for 316L surfaces and capturing two temperature peaks per layer. Through this monitoring, the system enabled targeted adjustments to scan strategies, laying the foundation for mitigating residual stress-driven distortion and reducing geometric deviations in complex parts. Xu et al. [154] proposed an online welding status monitoring method for T-joint double-sided double arc (DSDA) welding based on the fusion of welding current, arc voltage, arc sound, and weld pool images. The method uses a lightweight YOLO-L model for weld pool ROI detection, extracts feature through time-frequency analysis and CNN, and builds an ensemble learning model with KNN, RF, and LightGBM, achieving a welding status recognition accuracy of 98.538%.

The development of a quality prediction model based on multi-source integrated data provides a foundation for subsequent closed-loop control. For instance, by fusing sedimentation strategy images, process parameters, and temperature data, a multimodal DL model achieves temperature prediction and uncertainty quantification [147]. Herberger et al. [155] integrated RGB camera and microphone data, using an MLP model to predict LDED spacing in real time, thereby providing feedback for closed-loop adjustments.

### 3.6. Comparative Analysis of DL Applications in LDED and WAAM Processes

To further clarify the differentiated application logic of DL technology in two core DED processes—LDED and WAAM, Table 2 presents a comparative analysis across four dimensions which includes process characteristics, technical adaptability, outcome metrics, and data requirements. This comparison reveals the core differences and adaptation patterns in the application of DL technologies for these two processes.

From the perspective of process nature, LDED utilizes a highly focused laser beam, resulting in rapid dynamic fluctuations of the molten pool on a millisecond scale that are susceptible to powder spatter interference. Consequently, it necessitates DL models with high spatiotemporal resolution and high-precision sensors [138]. In contrast, WAAM employs a diffused arc as its energy source, leading to a larger molten pool size and stronger thermal inertia, with significant inter-layer heat accumulation. Therefore, it is more suited to lightweight models and conventional sensors, focusing on addressing issues like lack-of-fusion defect detection and inter-layer temperature control. This fundamental difference stems from the characteristics of the energy source and material form: the high focusing capability of the laser necessitates an LDED technical system that emphasizes “micro-scale dynamic capture”, while the diffusive nature of the arc and the continuity of the wire make WAAM more concerned with “macro-scale dimensional stability”.

In engineering practice, the application of DL in these two processes requires tailored technical approaches. LDED needs to address powder spatter occlusion through GAN-based image enhancement, combined with DRL-PPO algorithms to achieve rapid laser power adjustment. WAAM, on the other hand, can utilize a combined MFCC+CNN model to extract low-frequency acoustic features of the arc and employ DQN algorithms to optimize wire feed speed. Simultaneously, differences in data processing and model complexity provide guidance for technology selection: LDED requires multi-step acoustic denoising and MFCC-CNN for defect detection due to complex noise composition, while WAAM adopts simpler statistical analysis and K-means clustering for multi-layer process monitoring, leveraging more stable acoustic signals [138,139].

This comparison not only delineates the adaptation boundaries of DL technologies for the two processes but also offers directions for future research: for LDED, further optimization of high-frequency signal real-time processing efficiency is needed. For WAAM, efforts should focus on strengthening multi-parameter collaborative control to mitigate inter-layer thermal accumulation and deformation. Ultimately, these advancements will drive DED processes toward an intelligent development direction characterized by “process-model-equipment” synergy.

**Table 2:** Comparison of DL applications in LDED and WAAM processes.

Comparative Dimension	Laser Directed Energy Deposition (LDED)	Wire and Arc Additive Manufacturing (WAAM)	References
Process characteristics	(1) Small molten pool (<5 mm), fast dynamic fluctuations; (2) Strong interference from powder spatter; (3) High energy density prone to micro-cracks	(1) Large molten pool (5–20 mm), strong thermal inertia; (2) Low signal-to-noise ratio due to arc radiation; (3) Prone to lack-of-fusion defects	[4,15,21,30]
Applicability of DL methods	(1) Optical: CNN + Attention mechanism (sputter-resistant, segmentation accuracy ≥94.7%) (2) Thermal Signal: Conv LSTM (millisecond-level temperature prediction, error ≤5%) (3) Acoustic: Wave Net (100–200 kHz high sampling rate)	(1) Optical: Lightweight CNN (e.g., MobileNetV2, positioning accuracy ≥88%) (2) Thermal Signal: Basic LSTM (inter-layer temperature prediction, error ≤ 8%) (3) Acoustic: MFCC+CNN (44.1 kHz sampling rate)	[81,103,113,138,139]
Data acquisition approaches	(1) Optical: Coaxial hyperspectral camera (25-50 bands) (2) Acoustic: 100 kHz high-frequency microphone (3) Thermal: Long-wave infrared camera (frame rate ≥ 50 fps)	(1) Optical: Off-axis CCD camera (1-3 bands) (2) Acoustic: 44.1 kHz standard microphone (3) Thermal: Mid-wave infrared camera (frame rate ≥ 25 fps)	[102,103,124,138,149]
Predictive results	(1) Defect Detection: Porosity recognition accuracy ≥ 91.2% (2) Process Control: Laser power adjustment response time ≤ 50 ms (3) Quality Prediction: Molten pool depth MAE ≤ 0.02 mm	(1) Defect Detection: Lack-of-fusion recognition accuracy ≥ 89% (2) Process Control: Wire feed speed adjustment step size ≥ 0.1 m/min (3) Quality Prediction: Height consistency error ≤ 3%	[15,81,111,138,151]

## 4. DL-Based Control of DED Additive Manufacturing

### 4.1. Hybrid DL-Control

During the DED process, DL approaches are capable of extracting critical features—such as size, temperature, and defects—from multi-source data as control objectives, allowing for accurate recognition and quantification of these targets, and enabling integration with conventional PID-based control for closed-loop optimization. For instance, Liao et al. [156] developed a U-net-based deep learning-assisted process monitoring and dual closed-loop control framework, which, by enhancing edge feature extraction, enables precise identification of printing states and deposition layer widths, and, through coordination between the PID controller and the travel distance controller, markedly improves deposition uniformity under external perturbations. Li et al. [157] utilized DL to determine the dual-droplet transition distance in dual-wire AM and implemented PID control to improve the stability of the printing process. Xiong et al. [158] designed a fuzzy controller, achieving effective control over the dimensional accuracy of the manufactured components. Wang et al. [159] employed CNNs to extract weld bead width features and incorporated an ADRC algorithm, successfully mitigating the impact of strong disturbances. Nevertheless, while ADRC can handle the geometric profile of multi-layer weld beads, its effectiveness remains affected by welding speed, thermal accumulation, and overlap distance. DL’s ability to extract features is highly dependent on the quality and quantity of training data, and its generalization is limited under abrupt process variations or data-scarce conditions. While conventional PID-based methods provide stability and reliability, their linear nature limits their capacity to cope with the complex nonlinear dynamics inherent in AM.

### 4.2. RL-Based Control

DRL exhibits outstanding sequential decision-making ability when addressing complex, nonlinear, and multi-parameter coupled control tasks [160]. In the RL framework, an agent perceives the environment state, selects control actions based on its policy, and evaluates task performance via a reward function. Dharmawan et al. [161] proposed a model-based RL method that employs Gaussian process regression and a greedy strategy to optimize cumulative rewards, with experiments showing substantial enhancement in surface quality, near-net-shape geometric precision, and reduced material consumption. Mattera et al. [89] established an RL industrial control framework based on process reward functions, integrating reduced-order models and DDPG controller training, and enabled sim-to-real transfer through a specialized simulator, offering a comprehensive data-driven intelligent control approach. Schürmann et al. [162] introduced an anomaly-driven RL framework which automatically generates reward functions, removing dependency on manual rules and process assumptions, enabling dynamic coordination of multiple process parameters, and markedly improving the intelligence and adaptability of in situ control. DRL is capable of addressing high-dimensional nonlinear, multi-objective, and sequential decision tasks, features adaptive learning ability, and can optimize integrated performance via reward functions. Nevertheless, DRL involves lengthy training, potentially unstable convergence, and sensitivity to reward function design; deployment in real industrial settings demands high-fidelity simulations or substantial experimental data, posing challenges for transfer to actual operating conditions.

### 4.3. Model Predictive Control

Combining DL with MPC has emerged as a crucial approach to advancing the smart capabilities of manufacturing processes. Chen et al. [151] proposed a TiDE neural network-based synchronous multi-step MPC framework that leverages efficient temporal feature extraction and multi-step prediction to markedly enhance the responsiveness and optimization performance of real-time manufacturing decisions. Likewise, Li et al. [163] developed a GRU-GAN predictive control framework combining GRUs and GANs. In DED experiments, it validated melt pool temperature tracking and depth-constrained control, producing control signals that are smoother and more stable than traditional PID, highlighting the potential for efficient proactive control guided by digital twins. Other research, including Li et al. [164], used Actor–Critic agents to estimate adaptive PID gains, and Mezaache et al. [165] applied particle swarm optimization (PSO) to select welding parameters to minimize the heat-affected zone in GMAW, representing optimization-based control approaches similar to MPC. DL-MPC integrates DL’s high-dimensional feature prediction with MPC’s optimized decision-making, enabling the management of multivariable coupling and constraints for smooth and accurate control. Nevertheless, DL-MPC relies on the precision of the predictive model and demands high computational resources, and its control performance may be compromised under complex process conditions or significant disturbances.

### 4.4. Integration of DL and Digital Twin

Digital twins are virtual mappings of physical processes, entities, or systems that fuse real-time data from the physical object to achieve dynamic monitoring and closed-loop control of the actual system [166]. At its core, digital twins rely on two-way data exchange between the physical and virtual domains, enabling data-driven prediction, optimization, and decision-making [167]. The technology has seen broad applications across intelligent manufacturing, healthcare, and smart grids [168,169,170]. Typical digital twin systems comprise physical entities, virtual models, data, services, and connection interfaces [171], using full-lifecycle data to support process monitoring, smart control, and system optimization [172,173]. In AM, digital twins are required to characterize the process environment in real time to mitigate the dynamic effects of process disturbances on forming quality [174]. Integrating DL with digital twins enables the development of predictive models for real-time decision support. For instance, Chen et al. [151] incorporated sequential DL models into digital twin systems for online decision-making, and Coi et al. [175] developed a DL-driven temperature field simulation platform. Nevertheless, a critical shared challenge is the dependence on high-quality, large-scale datasets. Lu et al. [176] trained reinforcement learning models with over 3300 samples, while Li et al. [69] utilized more than 40,000 samples for a single material and component. Transfer learning and self-supervised learning can help mitigate data limitations to some extent [177], yet high-quality, large-scale data are still essential for model efficacy. Generative learning approaches can further address data constraints and facilitate virtual parameter testing, enabling system response simulation in digital twin environments for process and performance optimization [69].

Additionally, a product lifecycle includes four phases: design, manufacturing, operation, and end-of-life disposal. Presently, studies and implementations of DL integrated with digital twins primarily concentrate on the manufacturing and operational phases, most commonly in monitoring and control, optimization, and predictive maintenance, whereas applications in the design and disposal stages are still relatively limited [178]. By comparison, the fusion of DL and digital twins in the design and disposal phases remains at an early exploratory stage, offering significant room for advancement.

## 5. Limitations, Challenges and Future Directions

### 5.1. Limitations

The application of DL in DED process monitoring is constrained by data quantity and quality in practical applications. In engineering scenarios, the required labeled data for DED process monitoring tasks exhibits distinct hierarchical demands. For basic defect classification tasks, each defect category requires 500–2000 labeled samples to ensure model generalization. For instance, Liu et al. [81] developed an Image-Enhancement Generative Adversarial Network (IEGAN) for melt pool segmentation in DED additive manufacturing, using 22,000 valid thermal images, which enhances contrast with a CII of 1.1429, outperforming the original GAN. When the sample size drops below 500, the model’s recognition rate for rare defects decreases by 15–20% [30,111]. High-precision regression tasks require over 5000 samples to capture the nonlinear “process parameter-signal-quality” relationship. Ren et al. [128] developed an RNN-DNN model for thermal field prediction in LAAM, training it on 47,152 deposition status matrices from 100 one-layer cases with 6 scanning patterns, which achieved 98.09% accuracy and good adaptability to different scan paths. More complex spatiotemporal prediction tasks require tens of thousands of samples incorporating multimodal sensor data and temporal labels. Sajadi et al. [77] proposed a physics-informed Convolutional Long Short-Term Memory (PI-ConvLSTM) framework for real-time 2D temperature field prediction in metal additive manufacturing, achieving prediction errors below 3% for thin-walled parts and below 1% for cylindrical and cubic parts. The framework utilized 2760 experimental thermal images and approximately 16,000 simulation-derived input–output pairs per geometry. However, industrial environments face major barriers in obtaining large-scale labeled data. The primary challenge stems from exorbitant experimental costs. DED process consumables are prohibitively expensive, a single multi-layer deposition experiment consumes hundreds of grams of material, while equipment calibration requires hours of time, resulting in prohibitively high sample acquisition costs [138].

Data annotation demands specialized expertise, particularly for defect identification which combines optical microscopy, XCT scanning, and signal processing with DED process knowledge. For instance, annotating 50–100 μm micro-pores in molten pool images requires first validating defect authenticity through XCT scanning, then aligning with optical images—a single sample annotation process taking 1–2 h [13,29]. Furthermore, extreme process conditions like high-temperature splashing and unstable arcs are difficult to replicate, leading to insufficient rare samples such as hot cracks and spheroidization defects.

To address data scarcity, existing research has developed three practical technical approaches that can reduce annotation requirements by 30–80%. Transfer learning utilizes pre-trained models from large public datasets like imagenet and industrial time-series datasets, requiring only fine-tuning of the top-level network to adapt to DED scenarios. Ren et al. [128] trained a model using thermal history samples generated by finite element simulations (FEM) with varying scan paths and experimental samples, reducing thermal field prediction errors compared to pure experimental data. The approach combines simulation-driven data augmentation with finite element simulations or PINNS-generated physics-compliant synthetic data to expand the training set. For “no-simulation-data” scenarios like new alloys or processes, few-shot learning employs meta-learning or metric learning to train universal feature extractors with only 50–100 labeled samples.

### 5.2. Challenges

In aerospace applications, the DED process must comply with standards like EZ-SB-19-01 [179]. However, DED technology has not yet been fully integrated with these certification requirements. While AI can detect defects such as holes and micro-cracks in operations, establishing correlations with equivalent initial defect size (EIDS, formerly EIFS) remains challenging. The defect morphology in AM differs significantly from traditional manufacturing defects, requiring multiphysics coupling modeling to establish correlations. Simultaneously, AI monitoring of mechanical part acceptance criteria depends on specific usage scenarios. Since there are no universal standards for parts, one-size-fits-all acceptance methods should be avoided. For example, under high stress conditions, AI should strictly control porosity in aeroengine blades, while in low-stress scenarios, the requirements for the fuselage frame can be relaxed. Universal standards may lead to excessive rejection or false pass rates, thereby increasing production costs. Therefore, an optimized model integrating “context-specific characteristics, certification requirements, and AI threshold adjustments” is crucial. Current limitations in AI’s contextualized standardization require further research, and hybrid processes with multimodal data fusion technologies can provide valuable references for future breakthroughs.

Current DL models exhibit two fundamental interpretive limitations in DED monitoring. First, while data-driven models like CNN and LSTM can establish correlations between signals and defects, they fail to explain “why a specific signal corresponds to a particular defect type”, resulting in a disconnect from physical mechanisms. For instance, a CNN model achieving 88% accuracy in predicting non-welded defects through thermal imaging cannot specify “below what temperature gradient would cause non-welding”, making it difficult for engineers to accept for process parameter adjustments [5,77]. Simultaneously, complex models like GAN and diffusion models lack physically meaningful intermediate features, leading to ambiguous decision-making logic. When Yangue et al. [11] used the DDIM model to reconstruct molten pool images, the generated high-fidelity images assisted defect detection but failed to explain “which pixel features correspond to abnormal molten pool flow”, leaving model optimization directionless.

To address these challenges, a balance between interpretability and accuracy can be achieved through “physical mechanism embedding” and “model architecture optimization”. First, embedding DED process mechanisms as constraints into physics-aware neural networks ensures predictions align with physical laws. Secondly, integrating attention mechanisms into CNN and transformer models highlights correlations between key signal features and defects, enabling visual representation of attention dynamics. Asadi et al. [103] demonstrated in their YOLOV8S molten pool segmentation model that “melt pool edge irregularity” contributed 62% to non-welding defects through attention heatmaps, leading to optimized laser scanning speeds that reduced non-welding rates. Third, decomposing complex models into “signal preprocessing, feature extraction, and quality mapping” modules allow hierarchical decision-making with interpretable outputs. For instance, in multi-sensor fusion systems, optical modules generate “molten pool aspect ratio” while acoustic modules produce “energy distribution in the 150 kHz frequency band”, with final process status determined through weighted fusion. Engineers can pinpoint root causes through anomaly detection in individual modules [145]. Beyond interpretability, DL models also face real-time performance and hardware compatibility challenges. Industrial DED equipment requires inference latency of ≤100 ms for monitoring models, whereas complex models like 3D CNNs often incur 200–300 ms latency on edge controllers, necessitating optimization through techniques such as model quantization. Regarding hardware compatibility, most DL models rely on GPU computing power, while industrial field devices are predominantly embedded systems with<10 TFLOPS processing capacity, requiring lightweight models.

### 5.3. Future Directions

Through analysis of 270 selected studies, we identified the primary bottleneck hindering DL development in DED as insufficient reproducibility and comparability of results. This issue stems from three core limitations in current research: First, the lack of standardized datasets for monitoring deep melting deposition processes. Most studies rely on self-collected datasets highly dependent on specific experimental conditions, such as equipment models, material types, and process parameters. For instance, Liu et al. [81] constructed a melt pool image dataset for laser deep melting deposition using a customized laser powder supply system, while Chen et al. [138] collected acoustic signal datasets via a robotic LDED platform. These datasets vary in sensor resolution, sampling frequency, and defect marking standards, making cross-study model validation unfeasible. Only a limited number of studies have publicly disclosed their datasets, and none comprehensively cover all deep melting deposition scenarios, leading to “data silos” that constrain DL model generalization. Secondly, inconsistent testing protocols for model evaluation. Existing studies employ non-uniform experimental setups when validating DL models. In defect detection tasks, some studies use offline datasets for static evaluation, while others employ real-time process feedback in in situ testing, resulting in divergent performance metrics. Thirdly, the metrics used to evaluate DL models in defect detection are diverse and ambiguous. The reviewed studies employed over 20 metrics to assess model performance, with significant inconsistencies even for the same task. In defect classification, common metrics include accuracy, F1 score, and AUC-ROC. However, some studies only report accuracy, while others focus on F1 score to address class imbalance issues. For regression tasks like melt pool depth prediction, metrics range from MAE to RMSE and R^2^, making direct model comparisons challenging. For example, Ren et al. [128] reported RMSE using an RNN-DNN model for thermal field prediction, while Sajadi et al. [77] used a PINN model with MAE. These metrics cannot be directly compared to determine which model performs better, as they reflect different aspects of prediction errors.

To address the aforementioned issues of repeatability and comparability, this study proposes three actionable solutions, which will serve as a foundation for future DED standardization research. First, establish an open-access, multi-scenario defect detection benchmark dataset with unified specifications. These datasets should include (1) multimodal sensor data synchronized with high temporal precision; (2) detailed metadata containing device parameters, material properties, and environmental conditions; and (3) standardized defect labels validated through non-destructive testing. Secondly, establish standardized testing protocols for DL model evaluation in DED. Tailored to different DL application tasks, these protocols should specify data partitioning ratios to prevent overfitting caused by arbitrary division [30], incorporate real-time processing latency requirements and interference simulation field test protocols, along with reproducibility standards. Third, unify evaluation metrics by task type for quantitative model comparison. For classification tasks, core metrics should include accuracy, F1 score, and AUC-ROC. For regression tasks, MAE and R^2^ should be mandatory indicators (MAE reflects absolute error, R^2^ indicates model explanatory power). For control tasks, metrics like control latency, parameter fluctuation range, and defect reduction rate should be reported. A unified metric framework enables direct model performance comparisons.

## 6. Conclusions

DED, a core branch of metal additive manufacturing, holds irreplaceable value in the fabrication, repair, and remanufacturing of core components for high-end equipment in aerospace and other sectors. Ensuring the stability and consistency of processing quality is a critical prerequisite for its realization of large-scale engineering applications. Traditional experience-based process optimization can no longer address the technical challenges of multi-physics field coupling and complex dynamic characteristics in the DED process, while DL technology, with its powerful feature extraction and nonlinear modeling capabilities, has emerged as a core driving force for advancing DED toward intelligent development. This paper systematically reviews the current application status of DL technology in typical DED processes. Studies have confirmed that DL serves as an effective approach for defect detection, process optimization, and closed-loop control, demonstrating significant advantages in the analysis of optical, thermal, acoustic, and other multi-signal modalities as well as multi-sensor fusion. Meanwhile, it provides diverse technical pathways for control scenarios such as hybrid control, RL-based Control, model predictive control, and digital twin integration.

Nevertheless, these DL-based technologies still face limitations in practicality, including constraints related to data quantity and quality, model interpretability, real-time performance requirements of processes, and hardware compatibility. Due to the varying demands for labeled data scales across different tasks and the difficulty in extracting large volumes of data in industrial settings, data acquisition remains challenging, with insufficient data quantity and quality. Although methods such as transfer learning and simulation-driven data augmentation can reduce the need for labeled data, they cannot completely resolve the issue of data scarcity. Additionally, poor model interpretability and the inability of real-time monitoring performance to meet industrial requirements further hinder the development of such technologies. In the future, the repeatability and comparability of data can be enhanced by establishing open multi-scenario benchmark datasets, formulating standardized testing protocols, and defining unified evaluation criteria, while aligning with industry certification standards to adapt quality criteria for different working conditions. With the advancement of intelligent manufacturing, technologies such as the internet of things, digital twins, and cloud computing can be applied to the monitoring and control of DED technology. DL will further achieve deep synergy with DED processes, equipment, and standards, providing more robust technical support for the high-quality and high-efficiency fabrication of high-end equipment and driving the continuous development of additive manufacturing toward intelligence, large-scale production, and high reliability.

## Figures and Tables

**Table 1:** The clustering of selected literature.

Cluster	Keywords Samples	Research Domain
Cluster 1 (red + green)	Additive manufacturing, direct energy deposition, porosity, spatter, temperature, melt pool, image processing	Additive manufacturing processes
Cluster 2 (purple + blue)	Neural networks, distortion, parameters, machine learning, convolutional neural network, computer vision	Artificial intelligence and deep learning
Cluster 3 (yellow)	Closed-loop control, geometry, height control, quality control, regression, simulation	Monitoring and Process Closed-Loop Control

## Data Availability

No new data were created or analyzed in this study. Data sharing is not applicable.
